# Seasonality in Biological Rhythms in Scandinavian brown Bears

**DOI:** 10.3389/fphys.2022.785706

**Published:** 2022-04-07

**Authors:** Alexandra Thiel, Sylvain Giroud, Anne G. Hertel, Andrea Friebe, Olivier Devineau, Boris Fuchs, Stephane Blanc, Ole-Gunnar Støen, Timothy G. Laske, Jon M. Arnemo, Alina L. Evans

**Affiliations:** ^1^ Department of Forestry and Wildlife Management, Faculty of Applied Ecology and Agricultural Sciences, Inland Norway University of Applied Sciences, Koppang, Norway; ^2^ Research Institute of Wildlife Ecology, Department of Interdisciplinary Life Sciences, University of Veterinary Medicine, Vienna, Austria; ^3^ Behavioural Ecology, Department of Biology, Ludwig-Maximilians University of Munich, Martinsried, Germany; ^4^ Scandinavian Brown Bear Research Project, Orsa, Sweden; ^5^ Norwegian Institute for Nature Research, Trondheim, Norway; ^6^ IPHC, University of Strasbourg, Strasbourg, France; ^7^ UMR7178, CNRS, Strasbourg, France; ^8^ Department of Surgery, University of Minnesota, Minneapolis, MN, United States; ^9^ Department of Wildlife, Fish and Environmental Studies, Swedish University of Agricultural Sciences, Umeå, Sweden

**Keywords:** Brown bear (*Ursus arctos*), body temperature, circadian rhythm, heart rate, hibernation, lomb-scargle periodogram, multiday rhythms, activity

## Abstract

Biological rhythms, such as rhythms in activity and body temperature, are usually highly synchronized and entrained by environmental conditions, such as photoperiod. However, how the expression of these rhythms changes during hibernation, when the perception of environmental cues is limited, has not yet been fully understood for all hibernators, especially in the wild. The brown bear (*Ursus arctos*) in Scandinavia lives in a highly seasonal environment and adapts to harsh winter conditions by exhibiting hibernation, characterized by reduced metabolism and activity. In this study, we aimed to explore the expression of biological rhythms in activity, body temperature and heart rate of free-ranging brown bears over the annual cycle, including active, hibernation and the transition states around den entry and exit. We found that rhythms in physiology and activity are mostly synchronized and entrained by the light-dark cycle during the bears’ active state with predominantly diel and ultradian rhythms for body temperature, activity and heart rate. However, during hibernation, rhythms in body temperature and heart rate were considerably slowed down to infradian rhythms, influenced by the amount of snow in the denning area, whereas rhythms in activity remained diel. Rhythms in the transition states when bears prepared for entering or coming out of hibernation state displayed a combination of infradian and diel rhythms, indicating the preparation of the body for the change in environmental conditions. These results reveal that brown bears adjust their biological rhythms to the seasonal environment they inhabit. Rhythms in physiology and activity show simultaneity during the active state but are partly disconnected from each other during hibernation, when bears are most sheltered from the environment.

## Introduction

Animals living in the boreal geographical region experience a strong seasonality over the annual cycle, which is characterized by a high variance in light conditions, ambient temperature and food availability. Mammals and birds have adapted to these changes in environmental conditions by showing seasonal phenotypic plasticity. These adaptions help to synchronize the animal’s phenology with the environment and to allow optimizing of the timing of important life-history events.

In mammals this temporal organization is internally controlled by the interaction of different regions in the brain, such as the suprachiasmatic nucleus (SCN), the pituitary gland and the hypothalamus (Dardente et al., 2019) but can also be entrained by the environment (Golombek & Rosenstein, 2010; Myung et al., 2015). The field of chronobiology typically distinguishes the following biological rhythms-circadian/diel rhythms, which are approximately 24 h long, ultradian rhythms (<24 h) and infradian rhythms (>24 h) (Orem, 2005). The change in photoperiod throughout the year is considered the strongest cue (“*Zeitgeber”*) for animals to time life-history events, as it is a very predictable environmental signal (Bradshaw and Holzapfel, 2007), which enables the animals to prepare for annually changing conditions. The light-dark cycle allows for synchronizing of their activity, behavior and physiology with the 24 h cycle and entrains e.g., diel rhythms. Helm et al., 2017 emphasized the importance of integrating the fields of chronobiology and ecology into a joint research approach to overcome obstacles and understand the extent and regulation of temporal plasticity.

Studies, focusing on time-keeping of animals’ activity have often been conducted in regions with highly seasonal environments, such as the Arctic and Sub-Arctic (Swade and Pittendrigh, 1967; Pohl, 1999; Arnold et al., 2018; Ware et al., 2020). A study on free-living Svalbard reindeer (*Rangifer tarandus platyrhynchus*) found that significant circadian rhythmicity persisted throughout most of the year, despite midnight Sun in summer and Polar Night in winter (Arnold et al., 2018). However, an earlier study on the same species found only weak evidence for circadian rhythmicity (van Oort et al., 2007). Yet, another study investigated activity rhythms on Svalbard ptarmigan (*Lagopus mutus hyperboreus*) and found that ptarmigans become arrhythmic in arctic summers (Reierth and Stokkan, 1998). A key determining factor whether animals retain a 24-h rhythm in the absence of light cycles may lie in the amount of food availability. van Beest et al., 2020 found that musk oxen (*Ovibos moschatus*) that foraged in areas of high food productivity became arrhythmic during mid-summer and continuous light conditions, meanwhile individuals with no access to high productivity forage remained rhythmic. In species with winter hibernation, such as Arctic ground squirrels (*Urocitellus parryii*), circadian rhythmicity of physiological functions, such as body temperature, may be lost completely until spring emergence (Williams et al., 2017). It is likely that a variety of interacting factors, such as energy balance, metabolic state and risk perception may contribute to the expression or lack of circadian rhythmicity and that the relevance of the contribution of these factors is species-specific (Hazlerigg & Tyler, 2019).

Studying rhythms in activity in wild animals has been a great contribution to the field of ecology, however the integration of rhythms in physiology has been less in focus. We here address this gap for the brown bear (*Ursus arctos*) by studying rhythms in body temperature, activity and heart rate in a wild population in Scandinavia.

Brown bears in Scandinavia hibernate in a natural den, such as an abandoned anthill, a rock cave or uprooted tree for up to 7 months a year and are active throughout the rest of the year (Curry-Lindahl, 1972). Body temperature, heart rate and activity start to decrease 13, 24, and 25 days respectively before entering the den and start to increase again 33, 63 and 10 days before exiting the den, respectively (Evans et al., 2016a). Females become sexually mature with 4 years of age (Zedrosser et al., 2013) and mate in late spring—early summer (Dahle and Swenson, 2003) but display delayed implantation, which results in the implantation of the embryo at the beginning of hibernation and birth of on average one to four cubs (McLellan and McLellan, 2015) 6 to 8 weeks later (Schwartz et al., 2003), still hibernating. After emergence from the den, bears adapt their activity patterns not only to food resources, but also temporally to anthropogenic disturbance in order to avoid encounters with humans (Ordiz et al., 2014, 2017). However, rhythms in activity and physiology have not been evaluated yet for the brown bear in Scandinavia. Some studies have started to explore the diel organization of grizzly and black bears (*Ursus arctos, Ursus americanus*) in North America under their naturally changing conditions throughout the year but also in an experimental set-up, including the hibernation phase. Hibernating grizzly bears, housed in constant darkness showed circadian rhythms in body temperature and activity with a period length close to 24 h, which could be entrained by a single light exposure (Jansen et al., 2016). Rhythms in body temperature of hibernating American black bears in captivity were dependent on the temperature in the artificial den and the body size of the bear and bears showed infradian cycles in body temperature (Tøien et al., 2015).

Here we study the physiology and activity of Scandinavian brown bears in the wild. We aimed to explore (Objective 1) how rhythms in activity, body temperature and heart rate vary throughout the year and (Objective 2) which endogenous and exogenous factors influence the presence of rhythmicity.

## Material and Methods

### Study Area

The study was carried out in south-central Sweden (61°N, 15°E), an area dominated by coniferous forest, consisting mainly of Scots pine (*Pinus sylvestris* L.) and Norway spruce (*Picea abies* H. Karst) (Moe et al., 2007) with only 10% of the landscape exceeding 750 m above sea level (masl). Even though the density of human population is relatively low (Ordiz et al., 2014), human activity increases substantially in autumn, due to berry and mushroom picking activities as well as the annual bear (21 August—15 October) and moose hunt (1st September—31st January) (Nellemann et al., 2007). Monthly minimum, mean, maximum ambient temperature (T_a_), snow depth and day lengths for the study area can be found in Supplementary Table S2.

### Study Population and Data Loggers

We captured 44 brown bears (27 females, 17 males, 14–233 kg, 1–23 years of age) by immobilizing them from a helicopter from April to June 2010–2020 according to an established protocol (Arnemo and Evans, 2017) and as part of an ongoing ecological study, the Scandinavian Brown Bear Research Project (SBBRP). All bears were equipped with a global positioning system (GPS) collar (Vectronic Aerospace GmbH, Berlin) with dual-axis acceleration sensors to monitor activity (Friebe et al., 2014). GPS positions were recorded every 60 min. We used the bear’s body mass from the last time the bear was captured, which happens annually in spring. However, some individual bears were captured in February and June instead.

Activity was measured as true acceleration six to eight times per second on two orthogonal directions. Values for each direction were averaged over a 5 min interval, resulting in values ranging from 0 to 255. We summed the values of both directions to represent overall activity, ultimately resulting in values from 0 to 510 (Gervasi et al., 2006).

Body temperature (T_b_) was measured by abdominally implanted temperature loggers (DST Centi; Star Oddi, Iceland), which were programmed to record T_b_ every 5–30 min (see Arnemo and Evans, 2017; Evans et al., 2016a for further details on the implantation procedure).

Heart rate (HR) was measured by subcutaneously implanted cardiac monitors (Reveal DX and XT; Medtronic Inc., Minneapolis, Minnesota, United States) by monitoring HR continuously using an electrocardiogram, which converted the mean R-R intervals (rate of a ventricular cycle) into HR, stored at 2 min average values (Medtronic, 2017; Laske et al., 2018). Details about surgical procedures are presented in Evans et al. (2016b).

We selected the bears with HR and activity, for which we also had T_b_ available at the same time, which resulted in a smaller sample size for HR (N = 11 bears) and activity (N = 40 bears).

The study was reviewed and approved by the Swedish Ethical Committee on Animal Research (Uppsala, Sweden; Dnr 5.8.18-03376/2020), the Swedish Environmental Protection Agency (NV-00741-18), the Swedish Board of Agriculture (#31-11102/12), the Norwegian Food Safety Authority (FOTS ID 19368) and the Norwegian Environment Agency (2018/3346).

### Data Preparation

We obtained T_a_ and snow depth data of all Swedish weather stations from the Swedish Meteorological and Hydrological Institute (SE-601 76 Norrköpping, Sweden). We used inverse distance weighted interpolation from the R package *gstat* (Pebesma, 2004) and the function *krige* to spatially interpolate daily mean, minimum and maximum Ta and snow depth to hourly GPS positions of each bear or the den location during hibernation based on the three closest weather stations. We calculated day length for each day and bear with the function *daylength* from the R package *geosphere* based on the mean latitude for the study area.

We defined female reproductive state during hibernation as either pregnant or non-pregnant based on T_b_ and activity profiles (Friebe et al., 2014). During the study period, 9 out of 15 (60%) adult female bears became pregnant. Pregnancy was later confirmed through observation of cubs during the annual monitoring in June. Based on these observations and the pregnancy status, we defined a status category and categorized a bear as either pregnant, solitary or accompanied by cubs. Except for bears followed from birth, aging was done by sectioning of the root of one of the first premolar teeth (Harshyne et al., 1998). We defined bears of at least 4 years of age as adults (Zedrosser et al., 2013). All bears younger than that were defined as subadults.

Unrealistic T_b_ (T_b_ < 30°C, Tøien, et al., 2011, Evans et al., 2016a; Evans et al, 2016b) values were excluded from analysis, as well as the 2 weeks of data following a capture and 3 days following known episodes of disturbance including the annual cub monitoring from helicopter and experimental approaches (Ordiz et al., 2013; Evans et al., 2016a)).

**FIGURE 1:**
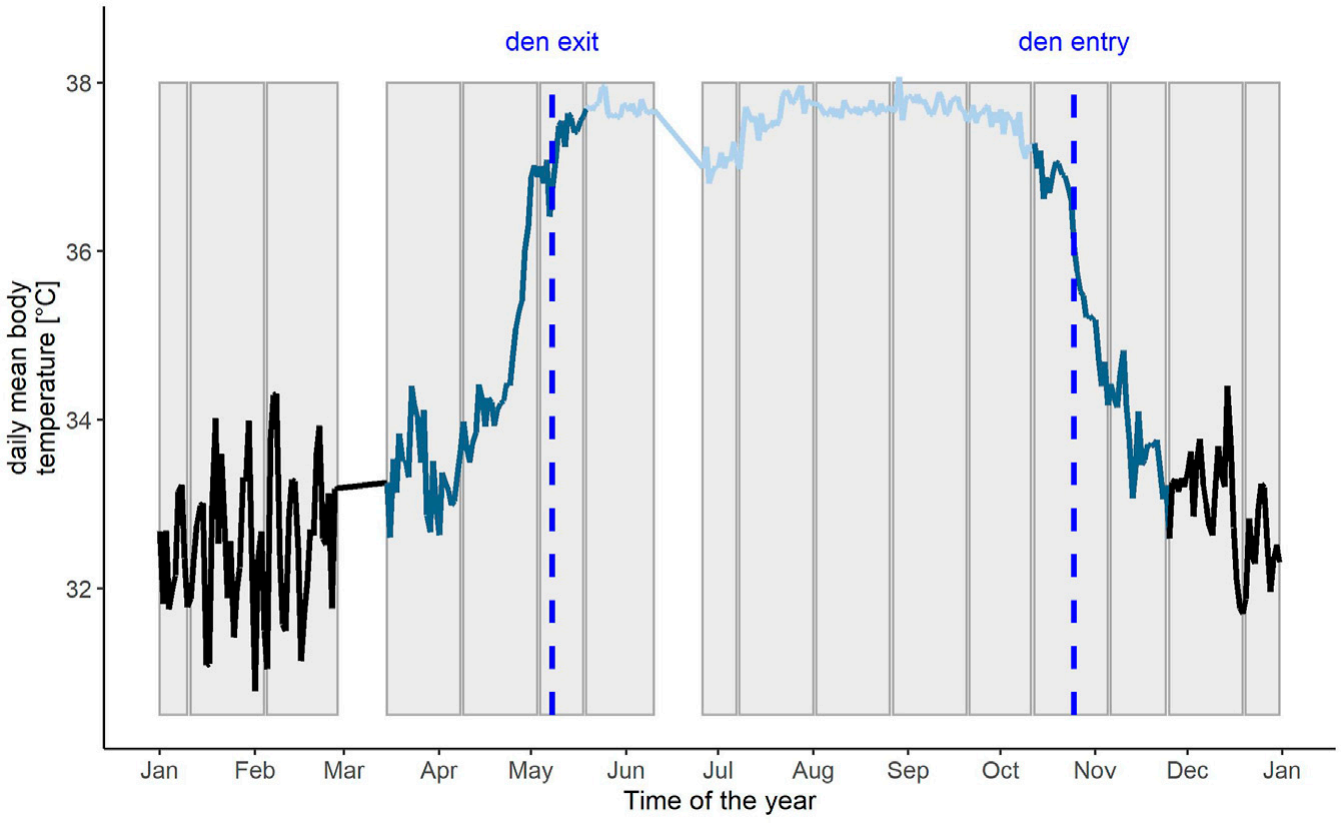
Example of the daily mean body temperature profile of a Scandinavian brown bear from January - December with the different time periods indicated as hibernation (black), transition (dark blue) and active phase (light blue). Den entry and den exit are shown as black dotted lines. The 15-25 days intervals, the biological rhythms analysis is based on, are indicated as grey bars in the background. White blocks represent data gaps due to capture events.

Based on the findings in Evans et al., 2016b we defined the following seasonal states within a bear’s year: 1) active, 2) transition around den entry, 3) hibernation and 4) transition around den exit state, depending on the variable in question (see Figure 1 for an example of T_b_). Den entry and exit are associated with a specific T_b_ in brown bears. Den entry was defined as the first day in autumn when daily mean T_b_ was <= 36.4°C for seven consecutive days. Den exit was defined as the first day in spring when daily mean T_b_ was >= 36.7°C (Evans et al., 2016a) for seven consecutive days. Based on the results of den entry and exit, we defined the transition state around den entry and den exit. Transition states around den entry and den exit vary in lengths for T_b_, HR and activity. For example activity starts to decrease on average 25 days before a bear enters the den and continues to decrease for 9 more days. T_b_ however starts to decrease only 13 days before den entry but continues to decrease for 30 more days, which leads to differences in lengths of the different seasonal states between the variables in question. The definitions for transition states of T_b_, HR and activity are presented in Supplementary Table S1 (modified from Evans et al., 2016a). In some cases bears changed dens during hibernation, due to known or unknown disturbances, and we included the new den location in the calculation for the environmental variables for the associated time span.

### Data Analysis

All analyses and visualization of the raw data were performed in R (version 4.0.3).

#### Detection of Biological Rhythms

We used Lomb-Scargle periodogram (LSP) analysis implemented in the R package *lomb* to investigate periodicity in T_b_, HR and activity of the bears throughout the year and different states. It is recommended to perform this analysis on at least ten periods (in our case days) in order to detect biological rhythms (Sokolove & Bushell, 1978). Since we also wanted to investigate the possibility of multiday rhythms, we chose a general time span of 25 days to search for periodicities but also included time spans of >= 15 days in order to be able to detect possible flexibility around transition states around den entry and den exit. We excluded irregular time spans, with data gaps >2 days, which originated from the exclusion of capture and disturbance periods. The beginning of a new state (active, transition, hibernation) determined the beginning of a new 25 days time span (Figure 1). We searched for periodicities within each time span and selected the highest peak detected by the LSP algorithm. We followed characterization of rhythms based on van Beest et al., 2020 and classified theses peaks as either ultradian (2–18 h), diel (18–36 h) or infradian (36–168 h) or, in the case of non-significant peaks as arrhythmic. The results of this analysis act as the response variable for the subsequent analysis. Additionally, we created actograms, thermograms and rasterplots of heart rate of the raw data for one representative 7 year old male brown bear with the *geom_tile* function in the R package *ggplot*.

#### Predictor Variables

We averaged environmental data (T_a,_ snow depth, day length) for each individual 15-25 days time span of each bear, resulting in a mean value over each individual 15-25 days time span. Furthermore, we obtained information about den location, den type and percent of open area of the den from the annual den monitoring program of the SBBRP. The set of possible predictor variables is presented in Table 1.

**TABLE 1:** Explanation of dependent and independent variables, which were available for inclusion in the statistical analysis.

Variable	Unit	Range/classification
Time period	categorical	active/hibernation/transition den entry/transition den exit
*Environmental predictor variables*		
Mean ambient temperature	°C	−15.4–19.1
Mean snow depth	m	0–0.63
Mean day length	hours	5.6–19.2
Direction of day length	categorical	increasing/decreasing
Den type	categorical	anthill/nest/rock/soil/uprooted tree
Open area	%	1–100
*Intrinsic predictor variables*		
Body weight	kg	17–233
Sex	categorical	Male/Female
Age class	categorical	adult/subadult
Status	categorical	solitary/pregnant/with cubs

#### Statistical Analysis

We fitted generalized linear additive mixed models to investigate (Objective 1) the probability a certain rhythm in T_b_, HR or activity is shown depending on the seasonal state of the bear and (Objective 2) which internal and external factors may influence the probability of a certain rhythm to be shown. In order to address the varying environmental effects throughout a year, investigated in the second objective, we split the data sets and investigated hibernation and active states separately.

We addressed both objectives in a Bayesian framework and analyzed them with the R package *brms* (Bürkner, 2017). We used multinomial logistic regressions to model the categorical response variable *Rhythm*, included the default flat priors of *brms* and fitted the models with 3 MCMC chains. We included continuous population-level effects as thin plate regression smoother terms to account for potential non-linear effects, allowing the whole term to shrink to zero (a linear effect) if appropriate.

For Objective 1, we were only interested in the effect of the different states (active, hibernation, transition den entry, transition den exit) on the response variable and did not set up a list of a priori candidate models. We ran three separate models for T_b_, activity and HR with 10,000 iterations, a warmup of 5,000 iterations, a thinning of 10 and adjusted the sampling parameters until we reached proper convergence of all models (Supplementary Table S7). We included the Bear ID as a group-level effect (Table 2).

**TABLE 2:** Set of a priori candidate models and model ranking based on Supplementary Tables S3-S6 for Objective 1 and 2 for T_b_, activity and HR datasets. All models for Objective 1 were fit with a group-level effect for the individual Bear ID. All models for Objective 2 were fit with a group-level effect for the individual Bear and Season.

Response variable *Objective1*	Predictor variable	Model number	Model ranking Body temperature	Model ranking Activity
Rhythm	Time period			
***Objective 2***				
*Hibernation*				
Rhythm	Null model	0	7	0
	s (Body mass)	1	9	4
	Age class	2	12	8
	Sex	3	3	7
	Status	4	8	3
	s (Snow depth)	5	1	6
	s (Day length)	6	6	1
	s (Ambient temperature)	7	4	2
	s (Open area den)	8	10	5
	Den type	9	15	9
	s (Open area den) + s (Snow depth)	10	2	10
	Den type + s (Snow depth)	11	14	12
	s (Open area den) + s (Snow depth) + Status	12	5	11
	Den type + s (Snow depth) + Status	13	11	13
	Den type + s (Snow depth) + s (Open area den)	14	13	14
*active state*			
Rhythm	Null model	0	9	8
	s (Body mass)	1	7	6
	Age class	2	5	11
	Sex	3	12	7
	Status	4	10	9
	Direction of Day length	5	11	5
	s (Day length)	6	2	2
	s (Ambient temperature)	7	6	12
	Snow depth	8	13	10
	s (Day length) + Status	9	4	4
	s (Ambient temperature) + Status	10	8	13
	s (Day length) * Direction of Day length	11	1	1
	s (Day length) * Direction of Day length + Status	12	3	3

For Objective 2, we ran the models with 4,000–5,000 iterations, a warmup of 2000 iterations and adjusted sampling parameters as presented in Supplementary Table S7. A group-level effect for the individual Bear ID and Season was included (Table 2).

For Objective 2 we set up a list of a priori candidate models (Table 2) and used leave-one-out cross-validation values (LOO) to select the most accurate model (Vehtari et al., 2017). In case several models were within a theoretical expected log pointwise predictive density (elpd) of <4, we used stacking of predictive distributions to evaluate which of these models has the highest probability to be the most accurate model (Yao et al., 2018) and chose to present the model with the highest stacking weight. We excluded observations where no information about environmental conditions was available. Due to the low sample size for the HR data set (N = 177 for the whole year) we were unable to test Objective 2 for HR.

We checked the models’ convergence and stability visually, checked that Rhat values did not exceed 1.01 (Vehtari et al., 2021) and for appropriately high Effective Sample Size (ESS) values around 1,000.

## Results

### Objective 1

#### Detection of Variation of Rhythms in Activity, T_b_ and HR throughout the Year

We used three different data sets (Activity, T_b_, HR) in our analysis for biological rhythms in free-ranging Scandinavian brown bears. We included 40 bears and 425 individual 15–25 -day time spans for activity, 44 bears and 693 individual 15–25 -day time spans over four different states (active, hibernation and two transition phases) for T_b_, and 11 bears and 123 individual 15–25 -day time spans for HR. An overview of how the proportions of rhythms vary over the year (divided in 15-25-days time spans) is presented in Supplementary Figure S2.

### Activity Data Set

**FIGURE 2:**
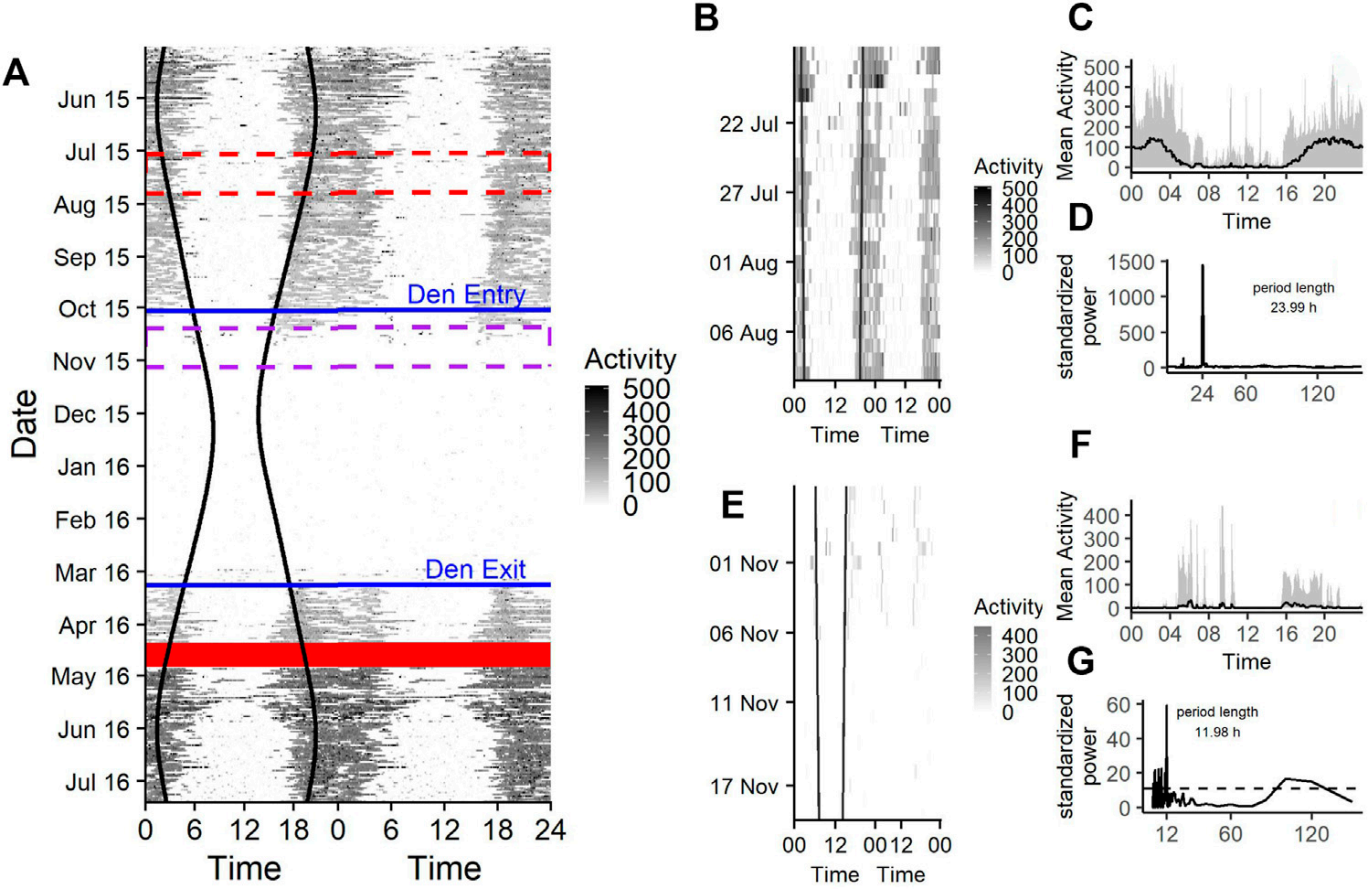
**(A)** Doupleplot actogram of the raw activity data of a 7 year old male Brown bear (*Ursus arctos*) with measurements from 15th May 2015–31st July 2016. The *x* axis represents the time of day x (left) and x + 1 day (right) from midnight to midnight and the *y* axis represents the time of the year in days. Activity is visualized with a color gradient from white to black with increasing activity values. Sunrise and sunset time are represented as black vertical lines, the excluded 14 days after capture (29th April 2016) are shown as a red ribbon and the den entry and den exit days are indicated as blue horizontal lines. The red and purple dashed rectangle are zoom illustrations for a 25-days time span during the active state **(B)** and hibernation **(E)**. The mean activity during the 25-days time spans over a day are shown in **(C)** and **(F)** and the corresponding Lomb-Scargle Periodograms and the period lengths for this time span are shown in **(D)** and **(G)**.

A representative actogram of the raw activity measurements is shown in Figure 2A, which shows a crepuscular and nocturnal activity during the bears’ active state. During midday and afternoon activity levels are low (Figures 2B,C), comparable to those during hibernation state (Figures 2E,F).

**FIGURE 3:**
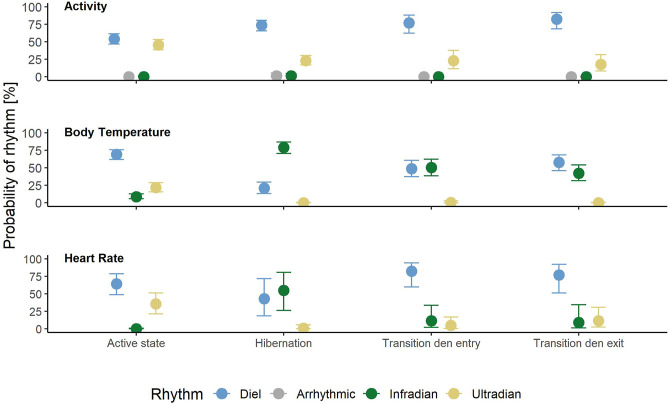
Probabilities of showing diel (blue), arrhythmic (grey), infradian (green) or ultradian (yellow) rhythms in body temperature, heart rate and activity in Scandinavian brown bears by time period (active period, hibernation, transition around den entry and den exit). Error bars indicate 95% credible intervals.

Rhythms in activity were either classified as diel, ultradian, infradian or arrhythmic. During the active state bears had a similar probability of being diel (54% credible interval CI [47–61%]), (Figure 2D, Figure 3) or ultradian (46% [CI 38–53%]) with negligible probabilities of arrhythmic or infradian rhythms. While hibernating, bears had the highest chance of expressing diel rhythms with 73% probability [CI 66–80%], followed by a 23% [17–30%] chance of being ultradian (Figure 2G, Figure 3). Infradian and arrhythmic rhythms were also present, but negligible. During the transition states bears had the highest probability of showing diel rhythms, followed by ultradian rhythms in activity (Figure 3). Mean period lengths for diel rhythms were about 24 h during all time periods for activity, whereas the mean ultradian period length decreased from 12 h during the active and transition states to 5 h during hibernation (Table 3).

**TABLE 3:** Average (mean), minimum (min) and maximum (max) period lengths [hours] and sample size of observations (n) and sample of individuals (n Individuals) of rhythms in activity, body temperature and heart rate in Scandinavian brown bears based on time period (active period, hibernation, transition state around den entry and exit). *Arrythmic rhythms, highest peaks in the Lomb-Scargle Periodogram without a significant signal.

Time period	Rhythm	Mean	min	Max	n	n Individuals
		Period length [h]	Period length [h]	Period length [h]		
ACTIVITY						
Active state	Diel	24	23.1	25	109	32
	Infradian	152	152	152	1	1
	Ultradian	12	12	12	84	33
Hibernation	Diel	24	20	24.7	130	32
	Arrhythmic*	5.4*	2.2*	12*	10	5
	Infradian	126.4	85.7	150	4	4
	Ultradian	5.1	2	12.4	55	18
Transition around den entry	Diel	24	23.1	25	34	25
	Ultradian	12	12	12.2	10	9
Transition around den exit	Diel	24.1	22.6	25	36	23
	Ultradian	12	12	12	7	7
BODY TEMPERATURE						
Active state	Diel	24.2	20.7	35.3	221	42
	Infradian	106.7	37.5	151.8	35	21
	Ultradian	11.6	6	16.2	77	32
Hibernation	Diel	24	21	26.2	31	19
	Infradian	118.6	37.5	168	110	34
Transition around den entry	Diel	23.9	23.1	24	53	31
	Infradian	115	41.4	152	58	28
	Ultradian	12	12	12	1	1
Transition around den exit	Diel	24	24	25	58	29
	Infradian	124.8	46.1	150	49	23
HEART RATE						
Active state	Diel	24	24	24	39	8
	Ultradian	12	12	12	22	9
Hibernation	Diel	24	24	25	27	7
	Infradian	129	60	168	32	5
	Ultradian	2.6	2.6	2.6	1	1
Transition around den entry	Diel	23.9	23.1	24	25	9
	Infradian	153.3	120	160	6	2
	Ultradian	12	12	12	2	2
Transition around den exit	Diel	24.1	24	25.1	17	5
	Infradian	122.2	66.7	150	3	2
	Ultradian	12	12	12	3	3

### T_b_ Data Set

**FIGURE 4:**
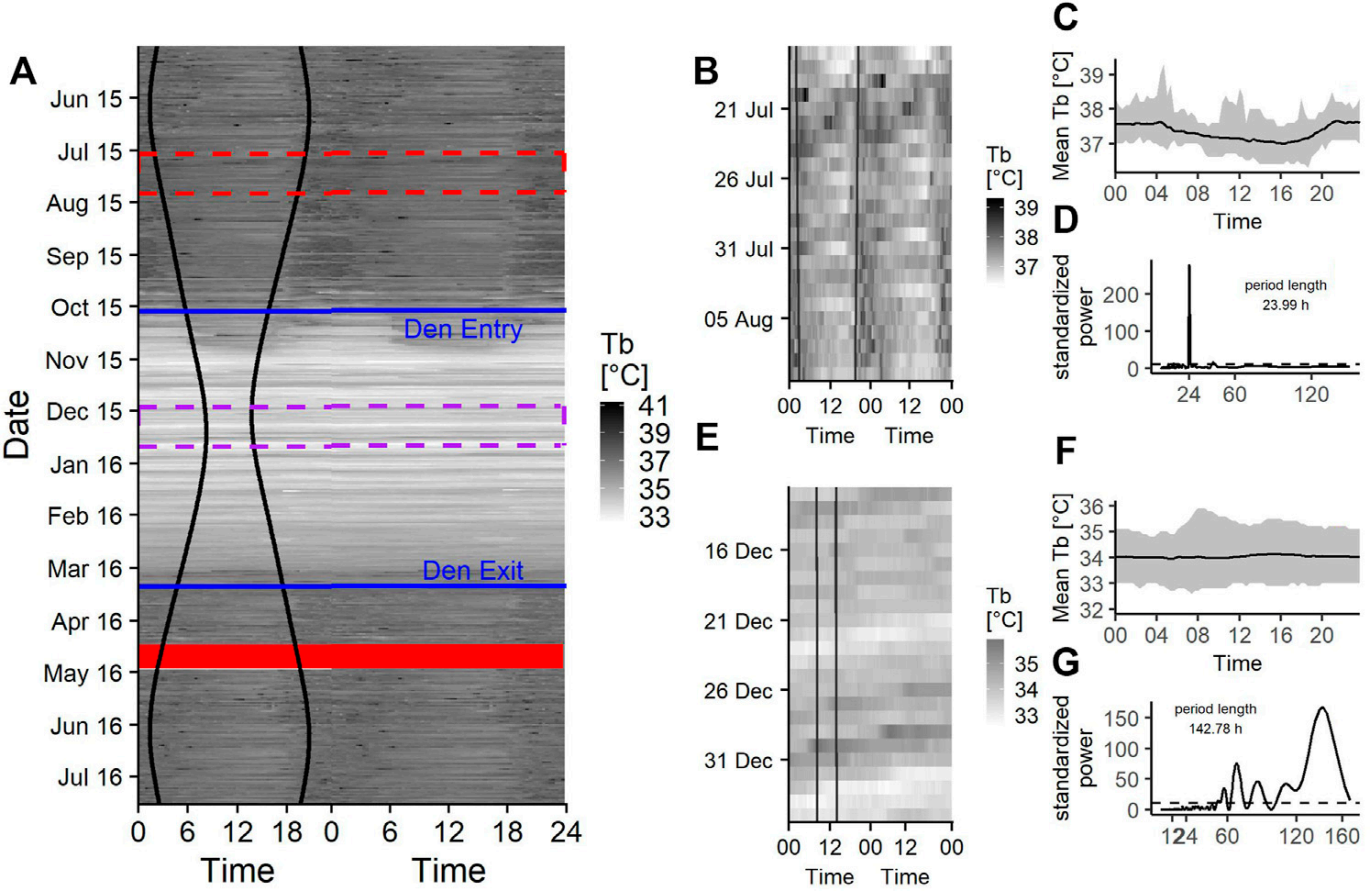
**(A)** Doupleplot thermogram of the raw body temperature (Tb) data sampled every 20 min of a 7 year old male Brown bear (*Ursus arctos*) with measurements from 15th May 2015–31st July 2016. The *x* axis represents the time of day x (left) and x + 1 day (right) from midnight to midnight and the *y* axis represents the time of the year in days. Body temperature is visualized with a color gradient from white to black with increasing body temperature values. Sunrise and sunset time are represented as black vertical lines, the excluded 14 days after capture (29th April 2016) are shown as a red ribbon and the den entry and den exit days are indicated as blue horizontal lines. The red and purple dashed rectangle are zoom illustrations for a 25-days time span during the active state **(B)** and hibernation **(E)**. The mean body temperature during the 25-days time spans over a day are shown in **(C)** and **(F)** and the corresponding Lomb-Scargle Periodograms and the period lengths for this time span are shown in **(D)** and **(G)**.

The seasonal changes in raw T_b_ data throughout the year are represented in Figure 4A, which shows the gradual adjustment of T_b_ around den entry and den exit dates and generally low T_b_ during hibernation (Figures 4E,F). During their active period in late spring, summer and early autumn, bears show a pattern of higher T_b_ from sunset to late morning and low T_b_ during midday (Figures 4A–C).

Rhythms in T_b_ were present as diel, ultradian and infradian rhythms. During the active state bears had a 69% [62–76%] chance of showing diel (Figure 3, Figure 4D) and 22% [16–29%] chance of showing ultradian rhythms in T_b_, respectively. During hibernation, this changed to bears having a higher probability of showing infradian rhythms (79% [CI 71–87%]), (Figure 3, Figure 4G) but only a 21% [CI 13–29%] chance of a diel rhythm. While transitioning between active and hibernation states bears had a similar probability of being diel or infradian, whereas ultradian rhythms were only negligibly present (Figure 3). For T_b_, the mean period length of diel rhythms was roughly around 24 h during active, hibernation and transition states. Ultradian rhythms during active and the transition states around den entry had a mean period length of around 12 h. Infradian rhythms during active, hibernation and transition around den entry state were around 5 days (Table 3).

### HR Data Set

**FIGURE 5:**
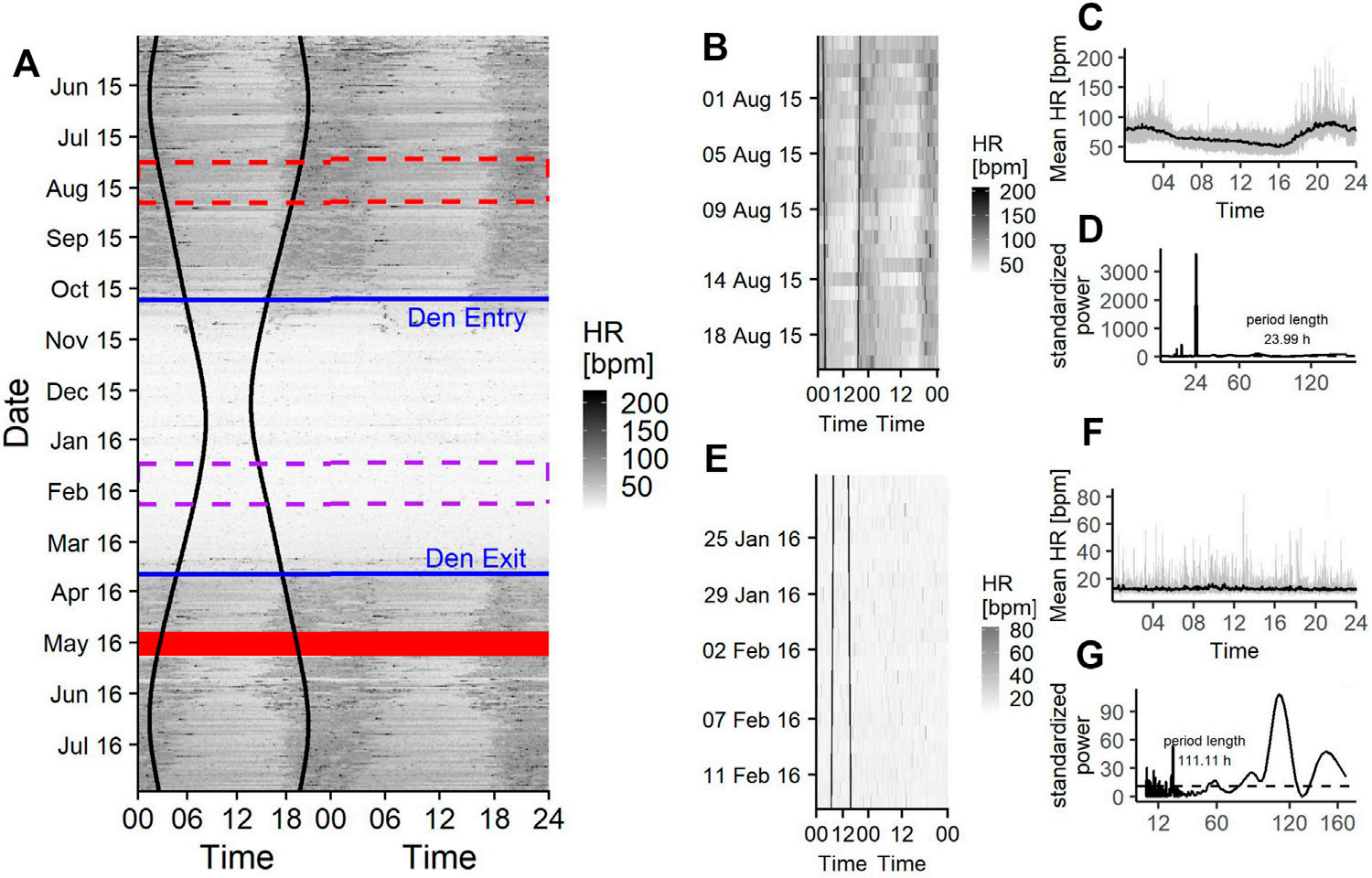
**(A)** Doupleplot rasterplot of the raw heart rate (HR) data of a 7 year old male Brown bear (*Ursus arctos*) with measurements from 15th May 2015–31st July 2016. The *x* axis represents the time of day x (left) and x + 1 day (right) from midnight to midnight and the *y* axis represents the time of the year in days. Heart rate is visualized with a color gradient from white to black with increasing heart rate values. Sunrise and sunset time are represented as black vertical lines, the excluded 14 days after capture (29th April 2016) are shown as a red ribbon and the den entry and den exit days are indicated as blue horizontal lines. The red and purple dashed rectangle are zoom illustrations for a 25-days time span during the active state **(B)** and hibernation **(E)**. The mean heart rate during the 25-days time spans over a day are shown in **(C)** and **(F)** and the corresponding Lomb-Scargle Periodograms and the period lengths for this time span are shown in **(D)** and **(G)**.

Similarly to the actogram, the rasterplot of the raw HR data shows a crepuscular pattern around sunrise and sunset and higher values at night time than during midday as well as a gradual adjustment of HR towards and out of hibernation (Figure 5A).

Rhythms in HR did not show any arrhythmic component but were diel, ultradian and infradian (Figure 3). During their active state, brown bears had the highest chance of displaying a diel rhythm (64% [49–78%]), (Figure 3, Figure 5D) in HR, followed by a 36% (21–51%) chance of being ultradian. During hibernation bears had similar probabilities for diel (44% [19–72%]) and infradian (55% [26–81%]), (Figure 3, Figure 5G) rhythms. However, credible intervals were wide and overlapping (Figure 3). Ultradian rhythms were negligible. When bears enter or exit the den, they have highest probabilities to display diel rhythms in HR (81% [60–94%] and 76% [51–92%], respectively). HR period lengths of diel and ultradian rhythms during active and transition states as well as hibernation were entrained to 24 and 12 h, respectively. The average period length of infradian rhythms during hibernation and the transition states were between 5.3 and 6.3 days (Table 3).

### Objective 2

#### Identification of Endogenous and Exogenous Factors Influencing the Presence of Rhythmicity

In the analysis for Objective 2, we split the data sets for activity and T_b_ into hibernation and active state. For activity, we included 31 bears and 142 individual 15–25-days time spans during hibernation and 37 bears and 179 individual 15–25-days time spans during the active state. For T_b_, we included 32 bears and 100 individual 15–25-days time spans during hibernation and 44 bears and 261 individual 15–25-days time spans during the active state.

### Activity Data Set

#### Hibernation

**FIGURE 6:**
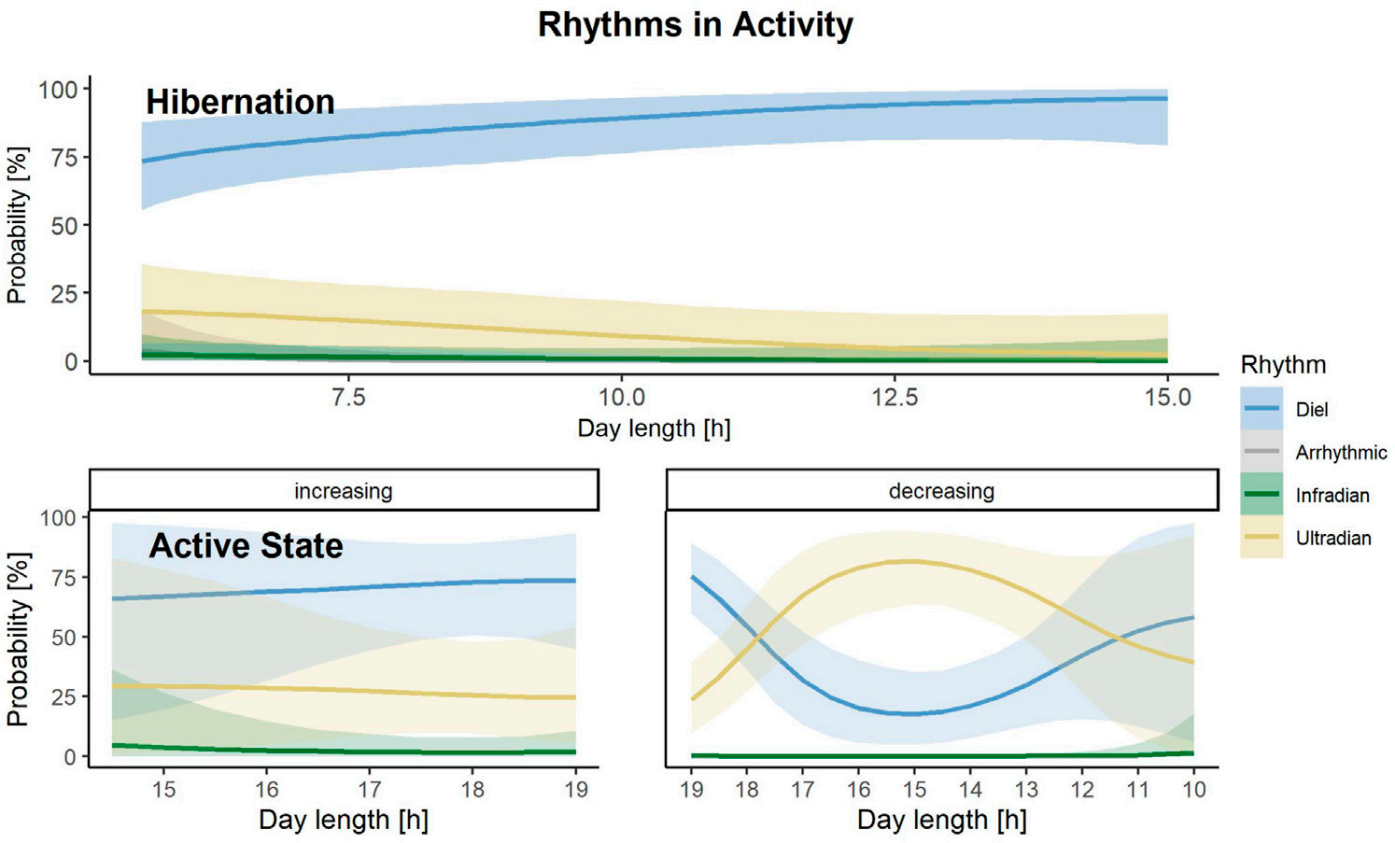
Prediction of the probability that either a diel (blue), infradian (green), ultradian (yellow) or arrhythmic (grey) rhythm in activity is displayed by Scandinavian brown bears during the active or hibernation state, depending on increasing or decreasing day lengths [h] during active state and increasing day length [h] during hibernation in the study area in south-central Sweden. Shaded areas indicate 95% credible intervals.

During hibernation, rhythms in activity were classified as diel, utradian, infradian and arrhythmic (Figure 3). The model, which performed best to predict the rhythms in activity during hibernation included day length only as a predictor (Supplementary Table S5). Overall, bears have a high probability of showing diel rhythms in activity during hibernation and this probability increases with increasing day length (Figure 6).

#### Active Phase

Rhythms in activity during the bears’ active state were classified as diel, ultradian and infradian (Figure 3). Four models within elpd <4 (Supplementary Table S6) best described rhythms in activity during that sate. However, all models had a similar predictive accuracy (difference in elpd <4), which is why we calculated the stacked weights of the models. The model with day length and the interaction with the direction of day length as a predictor had the highest stacking weight (w = 0.62). This indicates that the model including day length and the interaction with the direction of day length has a 62% chance of predicting the probability of rhythms in Activity accurately. When day length was increasing in spring until mid-summer, bears had the highest chance of displaying diel, followed by ultradian rhythms. When day lengths was decreasing from mid-summer until fall, this dynamic changed and bears had the highest probability of ultradian rhythms at day length between 17–13 h, which corresponds to August until mid-September in our study area. Infradian rhythms in activity were present but negligible (Figure 6).

### T_b_ Data Set

#### Hibernation

**FIGURE 7:**
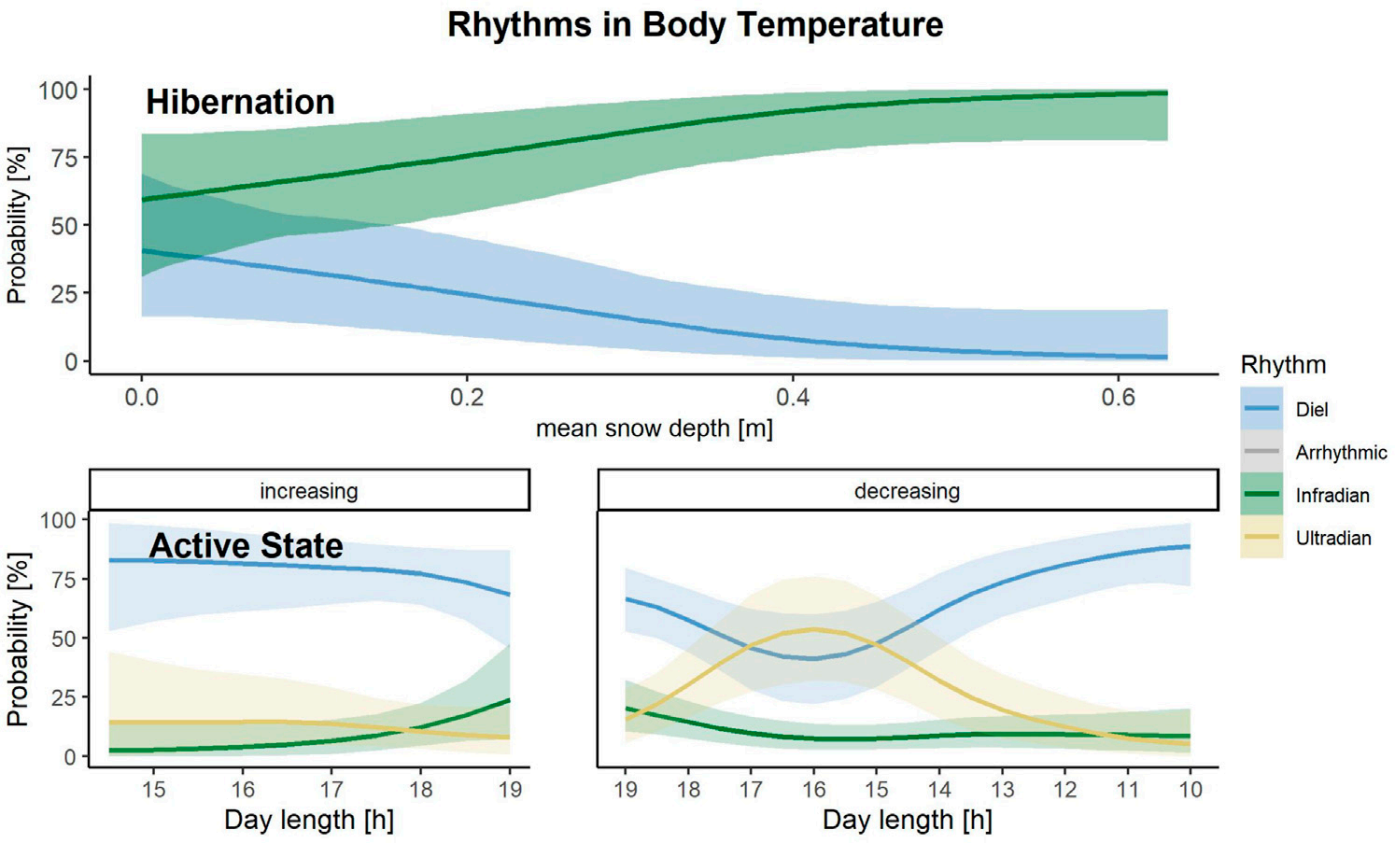
Predictions of the probability that either a diel (blue), infradian (green), ultradian (yellow) rhythm in body temperature is displayed by Scandinavian brown bears during their active or hibernation state, depending on increasing or decreasing day length [h] during active state and snow depth [m] during hibernation in the study area in south-central Sweden. Shaded areas indicate 95% credible intervals.

With the multinomial regression, rhythms in T_b_ were classified as diel and ultradian during hibernation (Figure 3). The most accurate model to predict which of the two rhythms in T_b_ is present during hibernation included the population-level effect snow depth. (Supplementary Table S3). The model, which included snow depth only had the highest stacking weight (w = 0.48). Increasing snow depth increases the chance of bears displaying infradian instead of diel rhythms (Figure 7).

#### Active Phase

Rhythms in T_b_ during the bears’ active state were classified as diel, ultradian or infradian (Figure 3). Four models performed similarly to predict the presence of these three rhythms in T_b_ (Supplementary Table S4, *N* = 261. The highest weight model included day length and the interaction with the direction of the day length as predictors (w = 0.57). During increasing day lengths, bears had the highest chance of showing diel rhythms. When day length decreased, and especially during day lengths of 17–15 h, which represents August in our study area, bears had equal probabilities of showing diel and ultradian rhythms. When day lengths decreased even more until the end of fall, bears showed predominantly diel patterns in T_b_ again (Figure 7). Probabilities for displaying infradian rhythms were generally low but increased with longer day lengths.

## Discussion

In this study, we clearly show that rhythms in T_b_ and activity of Scandinavian brown bears vary on a seasonal scale and are partly disconnected from each other during hibernation, when bears are most sheltered from the environment. During their active state in spring, summer and autumn, bears show mostly diel and ultradian rhythms in T_b_, activity and HR, dependent on the day length that is present. During hibernation, bears display predominantly infradian rhythms in T_b_, which is influenced by the amount of snow that is covering their den site, whereas rhythms in activity are mainly diel and HR shows similar probabilities for displaying both diel and infradian rhythms.

The change in photoperiod is the strongest *zeitgeber* wild animals use to time important life history events, such as reproduction, migration or hibernation in order to synchronize their life cycle with the seasonal environment (Bradshaw and Holzapfel, 2007). The daily light-dark cycle acts as the main factor to entrain an animal’s behavior and physiology to the 24 h day (Swade and Pittendrigh, 1967; Van Reeth et al., 1991; Weinert and Waterhouse, 1998). This was also apparent in our study, since the model including day length was the most accurate model to predict rhythms in both T_b_ and activity during the bears’ active state. Scandinavian brown bears in our study population exit their dens in a time period from end of march—beginning of May (Evans et al*.*, 2016b), which is accompanied by increasing day lengths from 12–16.5 h. Bears had the highest probability for showing diel rhythms in both T_b_ and activity (Figure 6, Figure 7) during that time of the year until mid-summer. A study on grizzly bears found that general activity is low from den emergence until mid-July (McLellan and McLellan, 2015) but in Scandinavia, this time of the year not only coincidences with peak mating season but also with bears predating mainly on moose calves, since berries are not yet available. Moose are generally most active around sunrise and sunset (Cederlund, 1989). Moose calves hide in the environment from predators (Zerbe et al., 2012) but when found by bears, are mainly killed during day time in Scandinavia (Rauset et al., 2012). This might explain that bears also had a 46% probability of showing ultradian rhythms in activity. The opportunistic foraging strategy of bears and overall flexible activity during that time of the year when photoperiod is changing rapidly, might explain the temporal high overlap in credible intervals between diel and ultradian rhythms in activity in our study. Generally, two rhythms, e.g., an ultradian and a diel rhythm, are not mutually exclusive and can occur simultaneously as a true combination of two rhythms, as seen in musk oxen when two significant peaks appear in the LSP (van Beest et al., 2020). In this study we analyzed only the rhythm with the highest peak in the LSP, i.e., the strongest signal, but want to emphasize that this does not exclude the possibility for two rhythms occurring simultaneously.

When the day length starts to decrease, from mid-summer to the end of July bears start to solely forage on berries (Hertel et al., 2016a) and do that mainly during crepuscular and night time hours, while resting during day light hours (Moe et al., 2007; Hertel et al., 2016b), avoiding human activity in the forest. This leads to an ultradian and diel pattern in activity, but also in T_b,_ with 12 and 24 h period lengths, as shown in our study. When day length decreases further from approximately 17–13 h, which represents August—September in our study area, the bears’ activity changes to predominantly ultradian rhythms. A similar pattern was obvious for Tb, however not as pronounced as for activity. Interestingly, all ultradian rhythms were expressed as 12-h rhythms with little variation around that. We do not have an explanation for the lack of variation but believe that the shift to nocturnal activity in order to avoid human activities in the forest may be causing the shift to ultradian rhythms during that time of the year. Generally, human activity is high at the end of summer/beginning of autumn in south-central Sweden due to berry and mushroom picking activities. Furthermore, from August 21st dogs are allowed off leash in our study area, which also coincidences with the first day of the annual bear hunt, followed by the annual moose hunt beginning 1st September. Ordiz et al., 2013 found lasting effects of experimental approaches of humans on Scandinavian brown bears, resulting in bears showing more nocturnal behavior after encounters with humans. These events may lead to a more ultradian but also generally flexible activity pattern in individual bears, depending on their exposure to these potential stressors (Hertel et al., 2017, 2021; Le Grand et al., 2019). Generally, during the bears’ active state, rhythms in T_b_ had a higher diel component than rhythms in activity. This indicates that these two rhythms are not perfectly synchronized even though they often are highly correlated under laboratory conditions in e.g., rodents (Refinetti, 1997, 1999; Weinert & Waterhouse, 1998). A study on Svalbard ptarmigan found a rise in T_b_ preceding the light-on signal as well as the rise in activity connected to the light-on signal, suggesting an anticipatory function through the diel system (Appenroth et al., 2021). The same may apply for rhythms in T_b_ in Scandinavian brown bears. Refinetti, 1994 found that at least 30% of the variations in the daily T_b_ rhythm is independent from the daily activity rhythm in golden hamsters (*Mesocricetus auratus*).

During hibernation, this strong *zeitgeber* photoperiod is no longer as available for Scandinavian brown bears as it is during their active phase. Snow depths of 15 cm reduce the detectable amount of light sharply and snow depths of 30–50 cm make light undetectable (Evernden and Fuller, 1972). Depending on the type of den, the bear starts to hibernate in, but also how the bear is positioned in the den, might lead to efficient isolation from the environment. Additionally, snow depths of 30 cm and more provide sufficient insulation in the subnivean space with stable temperatures around 0°C (Halfpenny & Ozanne, 1989; Marchand, 2014). Dens of bears in south-central Sweden are covered by 15–30 cm of snow from the beginning of hibernation on (November/December, Supplementary Figure S1). Snow depths of 20 cm coincidence with a change in probabilities of rhythms in T_b_ with bears having a higher probability of showing infradian rhythms the higher the snow depth becomes (Figure 7). The probability of a bear showing infradian rhythms was 79% during hibernation, indicating that the light-dark cycle is no longer entraining rhythms in T_b_ during hibernation. This is in line with results by Tøien et al. (2015) on American black bears, which were housed under captive conditions but also displayed infradian rhythms in T_b_. The authors concluded that the regulation of T_b_ cycles is not a passive process but rather active regulation. Even though bears do not show periods of arousal, such as other smaller hibernators (Tøien et al., 2015), bears might still need to actively regulate their T_b_ in order to maintain homeostasis and avoid T_b_ below a certain but not fixed threshold, which may lead to cardiac arrhythmia (Buresh et al., 2010). Depending on the insulating properties of the den and snow combination, period lengths of T_b_ may vary between 1.5 and 7 days with an average of 5 days (Table 3). In the study by Tøien et al. (2015) the period length of the T_b_ cycles decreased with colder temperatures in the artificial dens (T_den_). We were unable to assess T_den_ and assumed sufficient isolation from variation in environmental fluctuations, such as T_a_, when sow depth increased. A thick layer of snow may provide sufficient insulation, which would result in more stable temperatures in the den and potentially longer period lengths. However, a natural den of a hibernating brown bear is not a perfectly closed system and T_a_ and light might still be perceived, especially when snow depth is little and/or the den is not closed entirely.

This might contribute to our results in activity during hibernation, which showed period lengths around 24 h instead of infradian rhythms. This might be due to a true endogenously controlled circadian signal or entrainment by weak though sufficient light cues perceived through the snow cover, or a combination of both. Enright, 1970 stated that behavior is the result of a complex, interactive mixture of endogenous and exogenous factors. A study by Jansen et al. (2016) on American black bears, housed under constant darkness conditions found free-running diel rhythms in activity as well as T_b_, whereas bears in our study had only a 21% probability of displaying diel rhythms in T_b_ with average period lengths of 24 h. Harlow et al., 2004 hypothesized that hibernating American black bears display regular periodic muscle activity (as indicated by neck surface temperature) without accompanied elevated T_b_. They concluded that this facilitates the maintenance of muscular strength throughout the prolonged time of immobility during hibernation. Additionally, Jansen et al. (2016) found varying light intensities in dens of wild black bears and that the exposure to a single light signal had pronounced effects on the expression of diel rhythms in both T_b_ and activity, sufficient enough to entrain them to photoperiod. This might explain why probabilities of displaying a 24-h rhythm in T_b_ in our study on brown bears in Scandinavia was highest when snow depth was lowest and bears might have been exposed to light cues from the environment. For future studies, adding light sensors to the collars of hibernating brown bears could add valuable information about the environmental cues bears perceive in their dens. Additionally, Shiratsuru et al., 2020 found that older bears tend to choose den sites with a better fit to their individual body shape and better insulation properties, which in turn had a positive effect on their post-hibernation body condition index. The choice of den site and den cavity size/shape based on individual experience by the bear may have indirectly influenced the expression of biological rhythms, even though we did not find any effect of age and did not test for specific den cavity to bear-body shape-ratio.

We were unable to test Objective 2 for the HR data set, however we could get a picture of the probabilities of showing a certain rhythm in HR dependent on the time of the year. During their active and transition states, bears show similar probabilities in rhythms in HR as they show in rhythms in activity. Activity has been shown to have a stronger masking effect on rhythms in HR than on T_b_ (Gander et al., 1986). This might explain the similar expression of diel and ultradian rhythms in activity and HR during the bears’ active state. During hibernation, HR rhythms have similar probabilities of being diel and infradian, which seem to express a combination of masking effects of (low) activity and endogenous rhythms. Period lengths of infradian rhythms are in line with period lengths of T_b_ during hibernation, which indicates a similar endogenous control mechanism.

During the transition states around den entry and den exit, we analyzed biological rhythms based on 15–25-days time-spans, which is why den entry and den exit days may have fallen in between one of those periods. However, it is apparent that not only T_b_ and activity profiles are adjusting so that the bear is prepared to enter or exit hibernation by decreasing or increasing gradually (Evans et al., 2016a), but also their rhythms change (Supplementary Figure S2). This is likely regulated by a change in environmental condition, especially photoperiod, before den entry and change in the perception of this environmental cue due to den entry and exit.

This study shows the capability of brown bears to adapt their biological rhythms to the constantly changing environmental conditions they inhabit in Scandinavia. Rhythms in activity and physiology display similar period lengths and are entrained by the light-dark cycle during their active state but are partly disconnected during hibernation. Rhythms in physiology are considerably slowed down, whereas rhythms in activity remain diel. This sheds new light on the temporal organization of biological rhythms in free-ranging hibernators throughout the year but also opens up for questions about the endogenous control mechanisms that are responsible for the maintenance or loss of the diel organization in brown bears during hibernation.

## Data Availability

The raw data supporting the conclusions of this article will be made available by the authors, without undue reservation.
