# Supplementary material for: Seasonality in Biological Rhythms in Scandinavian brown Bears
Source: Front Physiol. 2022 Apr 7;13:785706. doi: 10.3389/fphys.2022.785706 (PMC9118031; doi:10.3389/fphys.2022.785706)
Supplement: Supplementary file 1 [file DataSheet1.docx]

Supplementary Material

| **Variable** | **Transition start** | **Transition end** | **Total** |
| --- | --- | --- | --- |
| *Body temperature* | | | |
| Transition den entry | 13 days before den entry | 30 days after den entry | 63 days |
| Transition den exit | 63 days before den exit | 10 days after den exit | 73 days |
| *Heart rate* | | | |
| Transition den entry | 24 days before den entry | 20 days after den entry | 44 days |
| Transition den exit | 33 days before den exit | 60 days after den exit | 93 days |
| *Activity* | | | |
| Transition den entry | 25 days before den entry | 9 days after den entry | 34 days |
| Transition den exit | 10 days before den exit | 46 days after den exit | 56 days |

Table S1: Definitions of transition phases around den entry and den exit for body temperature, heart rate and activity in Scandinavian brown bears based on Evans et al. 2016.

| Month | mean T_a_ | min T_a_ | max T_a_ | mean snow depth | min Snow depth | max snow depth | mean day length | min day length | max day length |
| --- | --- | --- | --- | --- | --- | --- | --- | --- | --- |
| January | -6.6 | -10.0 | -3.46 | 0.3 | 0 | 1 | 6.5 | 5.7 | 7.6 |
| February | -4.3 | -7.7 | -1.1 | 0.4 | 0 | 1.2 | 8.9 | 7.7 | 10.3 |
| March | -2.1 | -6.5 | 2.3 | 0.5 | 0 | 1.1 | 11.7 | 10.3 | 13.3 |
| April | 4.0 | -1.39 | 9.2 | 0.1 | 0 | 0.9 | 14.6 | 13.2 | 16.1 |
| May | 8.8 | 3.4 | 14.4 | 0 | 0 | 0.3 | 17.4 | 16.1 | 18.6 |
| June | 12.5 | 7.3 | 17.9 | 0 | 0 | 0 | 19.1 | 18.6 | 19.3 |
| July | 15.4 | 10.1 | 20.8 | 0 | 0 | 0 | 18.3 | 17.2 | 19.1 |
| August | 13.6 | 8.6 | 18.9 | 0 | 0 | 0 | 15.8 | 14.4 | 17.1 |
| September | 9.7 | 5.5 | 14.4 | 0 | 0 | 0 | 12.9 | 11.5 | 14.3 |
| October | 4.1 | 0.9 | 7.8 | 0 | 0 | 0.2 | 10.1 | 8.6 | 11.5 |
| November | -0.2 | -2.8 | 2.4 | 0 | 0 | 0.4 | 7.4 | 6.2 | 8.6 |
| December | -4.2 | -7.7 | -1.3 | 0.2 | 0 | 0.7 | 5.7 | 5.5 | 6.2 |

Table S 2: Monthly averaged (mean), minimum (min) and maximum (max) ambient temperature (T_a_ in °C), snow depth [meters] and day length [hours] in the study area in south-central Sweden from 2010-2020.

| Model Number  (according to Table 2) | difference  in elpd | difference  in standard error |
| --- | --- | --- |
| 5 | 0.0 | 0.0 |
| 10 | -0.6 | 0.6 |
| 3 | -1.1 | 2.7 |
| 7 | -1.5 | 2.1 |
| 12 | -1.8 | 2.1 |
| 6 | -1.9 | 2.3 |
| 0 | -2.1 | 2.2 |
| 4 | -2.3 | 2.4 |
| 1 | -2.7 | 2.3 |
| 8 | -2.7 | 2.3 |
| 13 | -3.1 | 2.7 |
| 2 | -3.3 | 2.3 |
| 14 | -3.5 | 2.7 |
| 11 | -3.5 | 2.3 |
| 9 | -3.6 | 3.1 |

Table S 3: Leave-one-out cross validation of candidate models to predict the rhythms in body temperature in Scandinavian brown bears during hibernation. Difference in elpd represents the difference in expected log pointwise predictive density

| Model Number  (according to Table 2) | difference  in elpd | difference  in standard error |
| --- | --- | --- |
| 11 | 0.0 | 0.0 |
| 6 | -0.7 | 3.9 |
| 12 | -1 | 1.5 |
| 9 | -1.3 | 4.1 |
| 2 | -5.5 | 6.7 |
| 7 | -7.4 | 4.3 |
| 1 | -7.6 | 6.2 |
| 10 | -8.6 | 4.5 |
| 0 | -11.6 | 5.7 |
| 4 | -12.2 | 5.9 |
| 5 | -12.3 | 5.2 |
| 3 | -13.1 | 5.8 |
| 8 | -13.2 | 6.1 |

Table S 4: Leave-one-out cross validation of candidate models to predict the rhythms in body temperature in Scandinavian brown bears during the bears’ active state. Difference in elpd represents the difference in expected log pointwise predictive density.

| Model Number  (according to Table 2) | difference  in elpd | difference  in standard error |
| --- | --- | --- |
| 6 | 0.0 | 0.0 |
| 7 | -5.6 | 3.8 |
| 0 | -6.8 | 3.3 |
| 4 | -7.7 | 4.1 |
| 1 | -8.1 | 3.4 |
| 8 | -8.6 | 3.5 |
| 5 | -8.6 | 3.7 |
| 3 | -9.7 | 3.7 |
| 2 | -10.4 | 3.4 |
| 9 | -11.1 | 4.7 |
| 10 | -11.2 | 4.2 |
| 12 | -11.9 | 4.7 |
| 11 | -14.1 | 5.8 |
| 13 | -15.2 | 6.7 |
| 14 | -15.7 | 6.0 |

Table S5: Leave-one-out cross validation of candidate models to predict the rhythms in activity in Scandinavian brown bears during hibernation. Difference in elpd represents the difference in expected log pointwise predictive density

| Model Number  (according to Table 2) | difference  in elpd | difference  in standard error |
| --- | --- | --- |
| 11 | 0.0 | 0.0 |
| 6 | -0.6 | 2.4 |
| 12 | -1.0 | 1.0 |
| 9 | -1.6 | 2.9 |
| 5 | -11.3 | 5.1 |
| 1 | -12.4 | 5.9 |
| 3 | -12.9 | 5.5 |
| 0 | -13.5 | 5.4 |
| 4 | -13.7 | 5.6 |
| 8 | -14.1 | 5.6 |
| 2 | -14.2 | 5.4 |
| 7 | -14.6 | 5.5 |
| 10 | -15.3 | 5.7 |

Table S6: Leave-one-out cross validation of candidate models to predict the rhythms in activity in Scandinavian brown bears during the bears’ active state. Difference in elpd represents the difference in expected log pointwise predictive density

|  | **adapt_delta** | **max_treedepth** |
| --- | --- | --- |
| **Objective 1** | 0.99 | 20 |
| **Objective 2** | 0.99 | 15-20 |

Table S7: Sampling parametes of models referring to Objective 1 and 2.


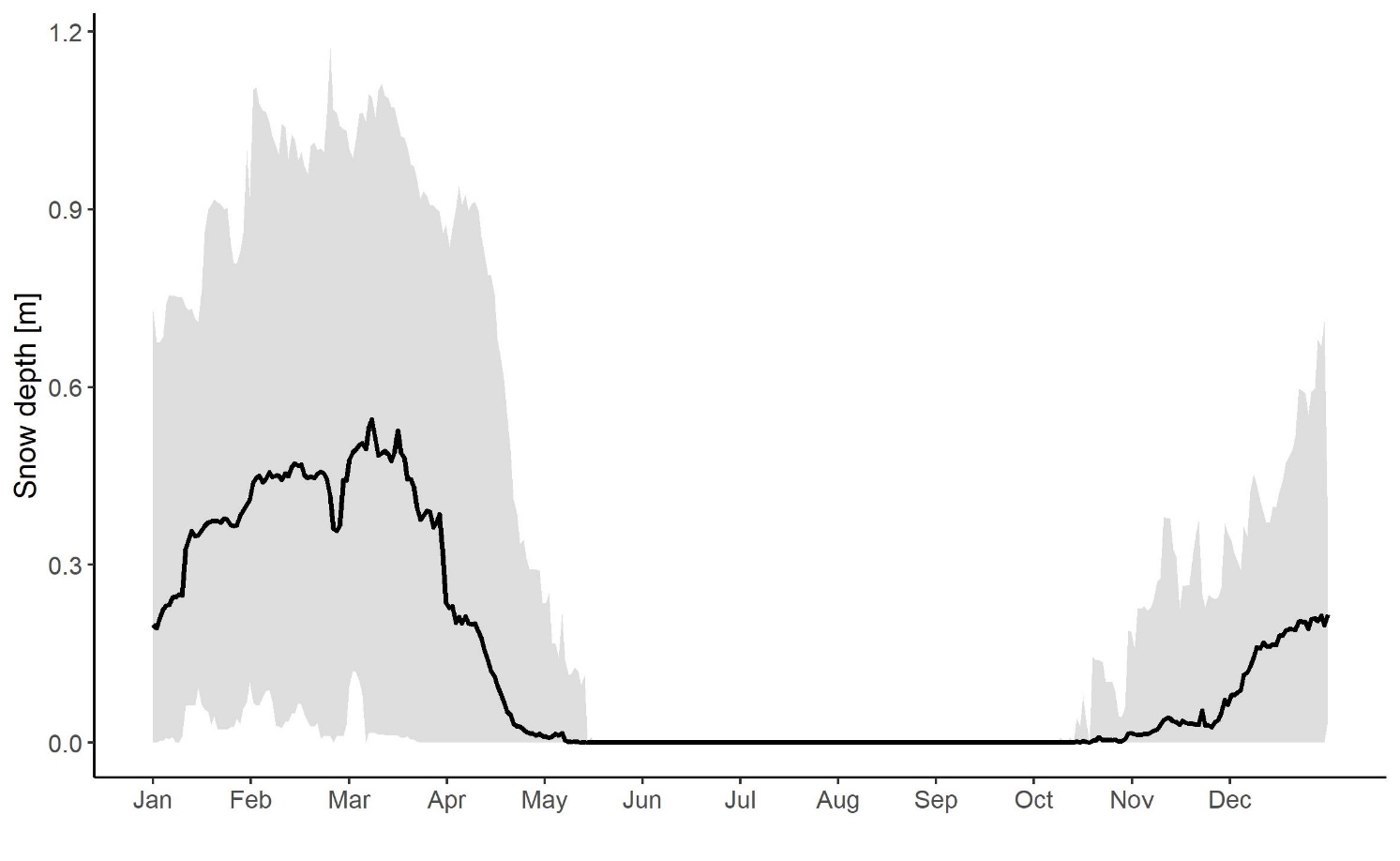


Figure S1: Daily averaged snow depth in study area in south-central Sweden. The black line represents the daily mean over all GPS positions and bears averaged and the ribbon around the daily mean represents the daily maximum and minimum of the daily mean Snow depth in meters.


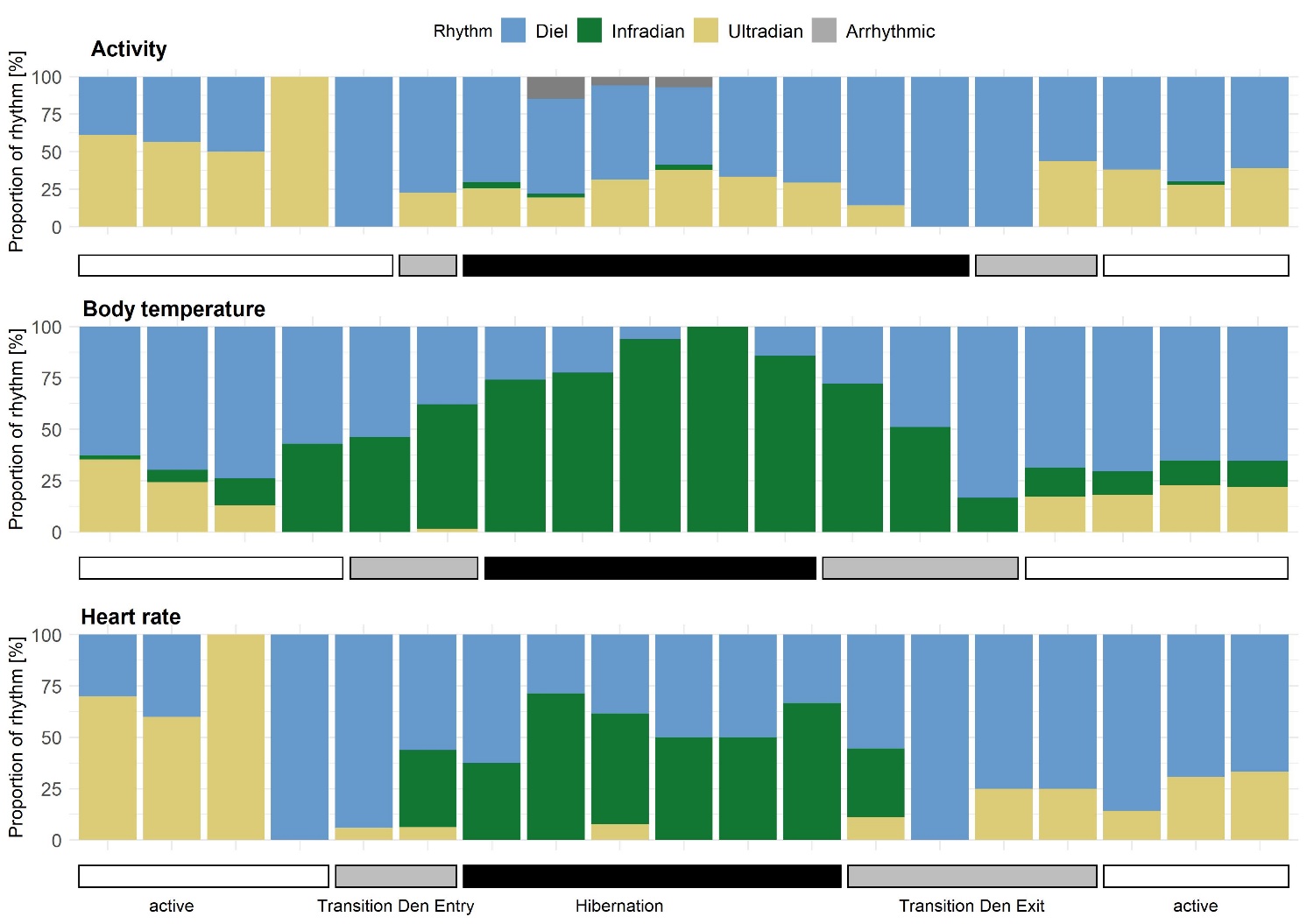


Figure S2: Proportion of rhythms in Activity, Body temperature and Heart rate (diel = blue, infradian = green, ultradian = yellow, arrhythmic = grey) displayed by Scandinavian brown bears for each 25 day time span during active (white bar), hibernation (black bar) and transition around den entry and exit phases (grey bars).
